# Molecular identification of *Fasciola* spp. (Digenea: Platyhelminthes) in cattle from Vietnam

**DOI:** 10.1051/parasite/2012191085

**Published:** 2012-02-15

**Authors:** S. Nguyen, S. Amer, M. Ichikawa, T. Itagaki, Y. Fukuda, Y. Nakai

**Affiliations:** 1 Laboratory of Sustainable Environmental Biology, Field Centre studies, Graduate School of Agricultural Science, Tohoku University 232-3 Yomogida, Naruko-onsen, Osaki, Miyagi 989-6711 Japan; 2 Department of Parasitology, Central Viet Nam Veterinary Institute km4 Dong De Street Nha Trang Viet Nam; 3 Department of Zoology, Faculty of Science, Kafr El Sheikh University Kafr El Sheikh 33516 Egypt; 4 Laboratory of Veterinary Parasitology, Faculty of Agriculture, Iwate University Ueda 3-18-8, Morioka 020-8550 Japan

**Keywords:** *Fasciola gigantica*, *Fasciola hepatica*, hybridization, parthenogenesis, ITS, COI, NDI, Vietnam, *Fasciola gigantica*, *Fasciola hepatica*, parthénogenèse, hybridation, ITS, COI, NDI, Vietnam

## Abstract

*Fasciola* spp. were collected from naturally infected cattle at a local abattoir of Khanh Hoa province, Vietnam, for morphological and genetic investigations. Microscopic examination detected no sperm cells in the seminal vesicles, suggesting a parthenogenetic reproduction of the flukes. Analyses of sequences from the first and second internal transcribed spacers (ITS1 and ITS2) of the ribosomal RNA revealed that 13 out of 16 isolates were of *Fasciola gigantica* type, whereas three isolates presented a hybrid sequence from *F. gigantica* and *Fasciola hepatica*. Interestingly, all the mitochondrial sequences (partial COI and NDI) were of *F. gigantica* type, suggesting that the maternal lineage of the hybrid form is from *F. gigantica*. No intra-sequence variation was detected.

## Abstract (fr)

Des individus de *Fasciola* spp. provenant d’animaux infectés d’un abattoir local de la province de Khanh Hoa (centre du Vietnam) ont été analysés morphologiquement et génétiquement. L’examen des individus au microscope n’a pas montré la présence de sperme dans les vésicules séminales, suggérant l’existence d’une reproduction parthénogénétique. L’analyse des séquences nucléaires ITS1 et ITS2 indique que 13 des 16 isolats correspondent à *Fasciola gigantica* et que les trois autres isolats seraient des hybrides entre F. gigantica et *Fasciola hepatica*. L’analyse des séquences mitochondriales COI et NDI de type *F. gigantica* suggère une origine *F. gigantica* de lignée maternelle des isolats hybrides. Aucune variation au sein des séquences pour les différents types n’a été détectée pour tous les marqueurs analysés.

Fasciolasis is a worldwide foodborne disease of both man and animals, with an impact likely to be higher in developing countries. Besides the burden for animal farm industry (Haseeb *et al.*, 2002; Phiri *et al.*, 2007; Mezo *et al.*, 2011), millions of people are estimated to be infected or at the risk of infection throughout the world (WHO, 1995; Keiser & Utzinger, 2009; Goral *et al.*, 2011). *Fasciola hepatica* is common in temperate zones especially in Europe, Americas and Australia, whereas *Fasciola gigantica* is the most prevalent species in tropical regions of Africa and Asia. Both *F. hepatica* and *F. gigantica* may overlap in subtropical areas (Mas-Coma *et al.*, 1999; 2005). Furthermore, hybridization/introgression phenomena might take place where both species coexist. *Fasciola* forms intermediate between *F. hepatica* and *F. gigantica* have been reported from Asian countries including Korea (Agatsuma *et al.*, 2000; Choe *et al.*, 2011), Japan (Itagaki *et al.*, 2005), Iran (Ashrafi *et al.*, 2006; Amor *et al.*, 2011), China (Peng *et al.*, 2009; Ai *et al.*, 2011) and Vietnam (Le *et al.*, 2008; Itagaki *et al.*, 2009) and as well as African countries including Egypt (Periago *et al.*, 2008, Amer *et al.*, 2011). Cytogenic peculiarities were reported in intermediate forms including different ploidies with no evidence of normal sperm production in most cases (Terasaki *et al.*, 1982; 2000). Molecular analysis of such individuals showed chimeric ITS sequences between the two species (Huang *et al.*, 2004; Itagaki *et al.*, 2005; Lin *et al.*, 2007; Le *et al.*, 2008). In some other cases, the nuclear DNA can be identical to one species whereas their mitochondrial DNA can be typical of the other species (Agatsuma *et al.*, 2000; Itagaki *et al.*, 2005; 2009).

High prevalence of animal and human fasciolasis has been reported in Vietnam (Anderson *et al.*, 1999; Holland *et al.*, 2000; Le *et al.*, 2008, Nguyen *et al.*, 2011). Ploidy related studies on specimens derived from cattle at Hanoi abattoirs indicated that these *Fasciola* might be hybrids between *F. hepatica* and *F. gigantica*. These hybrid forms seem to have originated in countries other than Vietnam (Itagaki *et al.*, 2009).

Little attention has been paid on molecular characterization of *Fasciola* sp. in the area of Khanh Hoa province, in central area of Vietnam. Therefore, the present paper aimed to extend the molecular profiling (based on sequences of the ribosomal ITS1 and ITS2 regions as well as partial mitochondrial COI and NDI genes) of Vietnamese *Fasciola* collected from cattle at Khanh Hoa province, Vietnam.

## Materials and Methods

Flukes were collected from livers of 14 naturally infected cattle brought to an abattoir in Khanh Hoa (central of Vietnam, 1,300 Km of Hanoi), Vietnam. Specimens were named after the location (Khanh) followed by the host (cattle = Ct) and the number of the isolate, in some instances more than one worm from the same animal were analyzed. Collected flukes were washed extensively in physiological saline, and individual worms were slightly pressed between two slides and fixed in 70% ethyl alcohol. Microscopic examination was carried out for inspection of the presence of sperm within the seminal vesicle.

Genomic DNA was extracted from a small portion of the posterior end of the fixed worms, using QIAamp DNA Mini Kit (Qiagen, USA) following manufacturer’s instructions. The DNA fragments of the ribosomal ITS1 and ITS2 regions and the partial mitochondrial COI and NDI genes were amplified utilizing the primer sets described by Itagaki *et al.* (2005). PCR reactions were done in 20 μl reaction volumes containing 50 ng of genomic DNA. The PCR mixture contained 1 × PCR buffer for KOD Plus Ver.2, 1 mM MgSO_4_, 0.2 mM dNTPs (each), 0.3 μM each primer, and 3.0 units KOD plus Polymerase (Toyobo, Osaka, Japan; final concentrations). Each PCR consisted of initial denaturation step at 95 °C for 5 min followed by 30 cycles of denaturation at 98 °C for 10 s, annealing at 56 °C (for ITS1 and ITS2) or 53 °C (for COI and NDI) for 35 s, and extension at 68 °C for 50 s; a final extension step consisting of incubation at 68 °C for 10 min was included. Products were subjected to electrophoretic separation using 1.5% agarose gels, stained with ethidium bromide, and visualized on a UV transilluminator.

PCR products were purified using Exonuclease I/ Shrimp Alkaline Phosphatase (Exo-SAP-IT^TM^; USB, Cleveland, OH, USA). Purified products were directly sequenced in a 20-μl reaction volume using the Big Dye^®^ Terminator v3.1 Cycle Sequencing Kit (Applied Biosystems Japan Ltd., Tokyo, Japan) on an automated sequencer (Applied Biosystems 3130XL Genetic Analyzer; Applied Biosystems Japan Ltd., Tokyo, Japan). Sequences were read using the ABI 3130 Genetic Analyzer software (SeqScap 2.1). The accuracy of data was confirmed by two-directional sequencing.

Maximum Likelihood method (ML) based on Tamura- Nei model (Tamura & Nei, 1993) with Invariant sites (I) were used to construct a phylogenetic tree based on the nucleotide sequences of COI. In the phylogentic tree all identical sequences were represented by a single one. Representative sequences were deposited in the database of the GenBank with the accession numbers of AB536905 to AB536916 for ITS1, AB536917 to AB536928 for ITS2, AB536893 to AB536904 for COI and AB536756 to AB536767 for NDI.

## Results and Discussion

Microscopical examination of the tested flukes detected no sperm cells in the seminal vesicles. Therefore, it is expected that these flukes reproduce parthenogenetically (WHO, 1995; Itagaki *et al.*, 2005). In contrast, Terasaki *et al.* (1982) and Itagaki *et al.* (2009) could detect normal and well developed sperm cells in *Fasciola* samples collected from other locations in Vietnam, along with the aspermic ones. Therefore, geographic differences may play a role in the distribution of spermic and aspermic *Fasciola* forms.

**Fig. 1.**
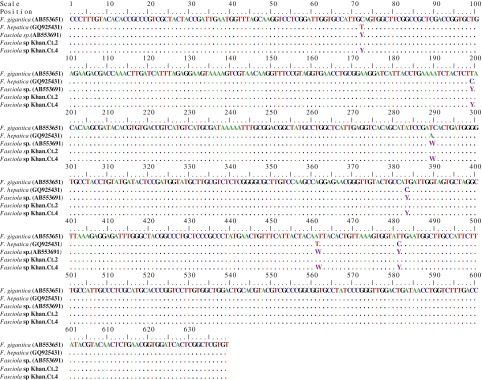
Alignment of ITS1 sequences of *Fasciola gigantica* (AB553651), *Fasciola hepatica* (GQ925431), *Fasciola* sp. (AB553691), Vietnamese *Fasciola* sp Khan.Ct.2 and *Fasciola* sp Khan.Ct.4.

**Fig. 2.**
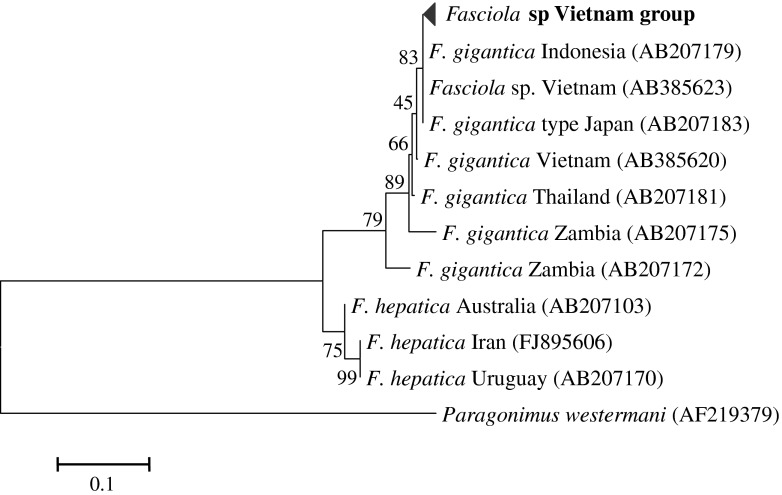
Phylogenteic relationship of *Fasciola* parasites based on sequences of COI. *Paragonimus westermani* (AF219379) was used as an outgroup. Evolutionary relationships of 12 taxa was inferred using the maximum likelihood method based on Tamura-Nei model (Tamura & Nei 1993) with Invariant sites (I). The percentage of replicate trees in which the associated taxa clustered together in the bootstrap test (2000 replicates) are shown next to the branches (Felsenstein, 1985). Phylogenetic analyses were conducted in MEGA5 (Tamura *et al.*, 2011).

In the present study, sequence analysis of ribosomal ITS1 of *Fasciola* worms revealed that 13 out of the 16 isolates were identical to the *F. gigantica* type, whereas three isolates (*Fasciola* sp. Khanh.Ct.1, 4 and 11.1) expressed both bases of *F. hepatica* and *gigantica* at all discriminating positions (Fig. 1), suggesting them to be intermediate form (Itagaki *et al.*, 2005). Similar results were obtained from sequence analysis of the ITS2 region that come in agreement with those of Choe *et al.* (2011). Moreover, the ITS2 of the isolate – *Fasciola* sp. Khanh.Ct.11.2 – proved to be the *F. hepatica* type, while the ITS1 region was the *F. gigantica* type. Moreover, the mitochonderial COI (Fig. 2) and NDI markers revealed that all isolates belonged to the *F. gigantica* type. In partial agreement with our results, Itagaki *et al.* (2009) categorized the *Fasciola* population from Vietnam to *F. gigantica* and the intermediate form of *Fasciola* based on the sequences of nuclear ITS1 and the mitochondrial COI and NDI genes. Moreover, Le *et al.* (2008) detected both *F. hepatica* and *F. gigantica* types among flukes derived from Vietnamese cattle, based on ITS2 with a *F. gigantica* mitochondrial background. Similarly, Nguyen *et al.* (2009) detected both *F. hepatica* and *F. gigantica* types among *Fasciola* worms from Vietnamese goats based on the ITS2 sequences, whereas the mitochondrial COI was identical to *F. gigantica*. In addition, Agatsuma *et al.* (2000) reported similar results for spermic specimens of *Fasciola* from Korea. In contrast, reports from China showed the existence of the three types of *Fasciola*: *F. hepatica*, *F. gigantica* and the intermediate form (Peng *et al.*, 2009; Ai *et al.*, 2011). Taken together, the results of this study and those of the abovementioned studies show a considerable level of genetic diversity in *Fasciola* populations in Vietnam.

Hybridisation and/or introgression involving both species of *Fasciola* may explain the presence of the intermediate forms recorded in the present study and in that reported by others (Agatsuma *et al.*, 2000; Le *et al.*, 2008; Nguyen *et al.*, 2009; Amor *et al.*, 2011). Nevertheless, Itagaki *et al.* (2009) showed that Vietnamese *Fasciola*, which were either diploid spermic (with capability of sexual reproduction) or triploid aspermic (parthenogenic reproduction) have the mitochondrial *F. gigantica* type, in spite of having identical nuclear sequences. Although we do not know about the ploidy of the here included specimens, the aspermic nature suggests that these flukes originated as a hybrid form undergoing clonal reproduction with no evidence of introgression. Itagaki *et al.* (2009) speculated that the aspermic *Fasciola* forms in Japan, Korea and Vietnam may have originated in other countries (and may also have a common origin) and spread rapidly into these countries with the infected host animals.
